# Evaluating the Importance of Leave Against Medical Advice (LAMA) and Associated Documentation in Patient Care

**DOI:** 10.1192/bjo.2026.11566

**Published:** 2026-06-30

**Authors:** Sidra Rasheed, Imtiaz Ahmad Dogar, IQRA AIN ALI, Habib Ur Rahman Yazdani, Asadullah Raoufy

**Affiliations:** Faisalabad Medical University, Faisalabad, Pakistan

## Abstract

**Aims:**

Leaving Against Medical Advice (LAMA) in psychiatric settings poses significant clinical, ethical, and legal challenges, particularly in low-resource countries like Pakistan. Inadequate documentation exacerbates risks to both patients and healthcare providers, yet compliance with institutional standards remains poorly evaluated. The aim of this audit is to assess adherence to LAMA documentation protocols and characterize the demographic and diagnostic profiles of psychiatric inpatients who left against medical advice.

**Methods:**

A cross-sectional clinical audit was conducted from February to July 2025, reviewing all LAMA cases (n=75) in the Psychiatry Inpatient Unit of Allied II (DHQ) hospital. Data were extracted from medical records, admission/discharge registers, and LAMA documentation using a structured checklist aligned with institutional standardoperating procedures. Compliance was evaluated across five core criteria: LAMA form completion, date/time documentation, dual-signature verification, LAMA register entry, and inclusion of a treatment summary

**Results:**

While 73.3% of cases documented the LAMA date and time, critical medico-legal elements were missing: 0% had completed the LAMA Notification Form or LAMA Register entry, and only 13.3% included dual signatures. Physician involvement was minimal (8.0%). Most patients were male (65.3%) with a mean age of 29.1 years. Substance use disorders (24.0%) and psychotic disorders (17.3%) were the most common diagnoses. Notably, reasons for LAMA were never documented.

**Conclusion:**

Profound documentation deficiencies undermine patient safety, legal protection, and quality of care. System-level interventions including standardized protocols, staff training, and structured exit interviews are urgently needed to improve LAMA management.

